# Metabolomics and Type 2 Diabetes: Translating Basic Research into Clinical Application

**DOI:** 10.1155/2016/3898502

**Published:** 2015-11-09

**Authors:** Matthias S. Klein, Jane Shearer

**Affiliations:** ^1^Faculty of Kinesiology, University of Calgary, 2500 University Drive NW, Calgary, AB, Canada T2N 1N4; ^2^Department of Biochemistry and Molecular Biology, Faculty of Medicine, University of Calgary, 2500 University Drive NW, Calgary, AB, Canada T2N 1N4

## Abstract

Type 2 diabetes (T2D) and its comorbidities have reached epidemic proportions, with more than half a billion cases expected by 2030. Metabolomics is a fairly new approach for studying metabolic changes connected to disease development and progression and for finding predictive biomarkers to enable early interventions, which are most effective against T2D and its comorbidities. In metabolomics, the abundance of a comprehensive set of small biomolecules (metabolites) is measured, thus giving insight into disease-related metabolic alterations. This review shall give an overview of basic metabolomics methods and will highlight current metabolomics research successes in the prediction and diagnosis of T2D. We summarized key metabolites changing in response to T2D. Despite large variations in predictive biomarkers, many studies have replicated elevated plasma levels of branched-chain amino acids and their derivatives, aromatic amino acids and *α*-hydroxybutyrate ahead of T2D manifestation. In contrast, glycine levels and lysophosphatidylcholine C18:2 are depressed in both predictive studies and with overt disease. The use of metabolomics for predicting T2D comorbidities is gaining momentum, as are our approaches for translating basic metabolomics research into clinical applications. As a result, metabolomics has the potential to enable informed decision-making in the realm of personalized medicine.

## 1. Introduction

Type 2 diabetes (T2D) is an increasingly widespread disease in both developed and developing countries [1]. By the year 2030, it is predicted that more than half a billion people worldwide will be affected by this disease [2]. Not only does T2D burden patients with numerous associated health complications such as cardiovascular disease, nephropathy, neuropathy, and retinopathy, but T2D is also a substantial strain on health care budgets [1].

Diagnosis of T2D is typically determined by fasting blood glucose and the oral glucose tolerance test that examines an individual's ability to dispose of a glucose load. Glycated hemoglobin (HbA1c), on the other hand, provides information on glucose management during the months preceding the initial testing. Despite these systematic measures, up to 60 percent of T2D cases are never diagnosed [3]. The reasoning behind this misdiagnosis, or lack of diagnosis, is likely due to the insensitivity of these assays at predicting prediabetic and diabetic threshold values [4]. Early diagnosis of T2D is extremely important, as early interventions might delay or even prevent full-blown disease [5–8]. T2D is rarely a static condition, but rather one that evolves and changes over time during the lifespan of the individual. Additionally, not all individuals are affected equally by the disease [9–11]. Indeed, clinical risk factors appear to cluster in certain individuals more than others and are often independent of body mass index (BMI) [12, 13]. It is these high-risk individuals that are most likely to benefit from early and aggressive lifestyle interventions. This underscores the need for enhanced diagnostic tools and the adoption of emerging technologies.

Considerable variation is also evident in the response of individuals to treatment as well as their susceptibility to diabetes-related complications [14]. This variability both in disease progression and treatment response emphasizes the need for additional tools for predicting disease progression and treatment success. This review examines* metabolomics* as a novel approach in achieving these goals.

### 1.1. Metabolomics

Metabolomics is the comprehensive characterization of metabolites in biological systems. The term metabolomics is similar to that of older technologies such as* genomics* (dealing with genes),* transcriptomics* (dealing with gene transcripts), and* proteomics* (dealing with proteins). The metabolome is comprised of small intermediary molecules and products of metabolism, including those associated with energy storage and utilization, precursors to proteins and carbohydrates, regulators of gene expression, and signalling molecules. Thus, the metabolome as the entirety of metabolites represents a real-time functional portrait of the cell or the organism. The metabolome is influenced by a plethora of factors, such as diet, lifestyle, medications, gender, and age. In this regard, metabolomics becomes a very powerful tool as it views the effects of pathological factors from vastly different origins in a single measurement.

Specific methods employed in the study of metabolomics include nuclear magnetic resonance (NMR) [15], gas chromatography mass spectrometry (GC-MS) [16], liquid chromatography mass spectrometry (LC-MS) [16], capillary-electrophoresis mass spectrometry (CE-MS) [17], and high performance liquid chromatography (HPLC) [18]. NMR measures differences in the magnetic properties of atomic nuclei, mass spectrometry measures differences in the mass and electrical charge of the metabolites, and chromatography distinguishes metabolites by differences in adhesion properties. While hyphenated MS methods have the advantages of high sensitivity, small sample volumes, and relatively low costs, NMR has a greater range of simultaneously detectable molecular species, as well as simple sample preparation and excellent reproducibility.

In all platforms, two fundamentally different approaches may be chosen:* targeted profiling* or* metabolic fingerprinting*. For targeted profiling, quantitative values for a preselected subset of metabolites are calculated. For accurate results, internal standards must be used to calibrate sample concentrations by adding reference substances of known concentrations. In mass spectrometry, for example, a stable isotope-labeled variant of the metabolite of interest is spiked into the sample. For NMR, a single concentration reference substance is added; however obtained values may need to be scaled by metabolite-specific individual calibration factors [19, 20]. The results are a quantitative measurement of the abundance of the metabolites in the sample. For metabolic fingerprinting, all peaks of a spectrum, also known as* features*, are used for statistical analysis. This usually includes a large number of unknown metabolites. After statistical analysis, the features of potential interest have to be assigned to their respective molecule using online metabolite databases such as the human metabolome database HMDB (http://www.hmdb.ca/) and the biological magnetic resonance database BMRB (http://www.bmrb.wisc.edu/). As researchers are constantly updating these databases with additional substances, the number of unidentifiable features is gradually declining. Fingerprinting has the benefit of yielding a more comprehensive view of the metabolome and may help identify previously unknown disease mechanisms; however targeted approaches often have a lower variance and, thus, larger statistical power to detect small effects.

As each platform (NMR, GC-MS, and LC-MS) provides unique information, with very little overlap in the detected metabolites, results often differ depending on the platform being used. One way to circumvent this issue is to employ more than one system for a given study. Although this approach increases the number of detected metabolites and provides a more comprehensive picture, it is not typically performed due to increased costs and a lack of equipment and expertise.

Samples most commonly analyzed via metabolomics include plasma, serum [21], and urine [22, 23], while other sample types such as cerebrospinal fluid [24] and saliva [25] are used less frequently. Metabolomics studies may also analyze tissue extracts [26]; whole tissue by means of magic angle spinning (MAS) NMR [27] and even living organisms may be analyzed by in vivo NMR [28]. Additionally, cell culture samples and supernatants may be analyzed [29].

At present, there are no commonly used, standardized protocols for sample collection and storage for metabolomics studies, a fact that may contribute to additional variation in metabolomic profiles. For example, some studies collect samples only from fasting participants, while other studies do not have this as a prerequisite. Significant concentration differences between the metabolites of plasma and serum prepared from the same blood sample have been found [30]. Although concentration differences have been found, plasma and serum metabolites correlate well with each other, indicating that both sample types allow accurate metabolomics measurements as long as serum and plasma samples are not compared to each other. While plasma showed higher over-time stability, serum allowed the quantification of more metabolites due to higher concentrations of selected metabolites [30]. Storage conditions are a crucial factor for metabolomics studies, especially for prospective studies, where samples may be analyzed many years after being collected. Studies found no differences between plasma that was frozen immediately versus plasma that was stored at 4°C for 8 [31] and 24 [32] hours before freezing, respectively. Likewise, no significant differences in metabolite profiles were found when comparing storage at −20°C and −80°C [33]. Plasma samples stored at −80°C for 13 to 17 years showed no influence of storage time on the metabolic profile [31]. All these studies indicate that the choice of sample type (serum or plasma) and the storage conditions may be a minor issue for metabolomics studies.

To examine metabolomic differences in response to T2D and PD in this review, we conducted a PubMed search for the following keywords: ((*diabetes type 2*)* OR* (*insulin resistance*))* AND* (*metabolomics OR metabolomic OR metabolite*) and filtered for human studies published within the last 10 years. This yielded 493 articles, which were subject to further screening. Only original articles were included, and studies that did not analyze serum or plasma were excluded. Only articles in English language were examined. These results were further filtered by including only papers dealing with diagnosis, prediction, prognosis, and translation of T2D.

## 2. Diagnosis and Prediction

Most T2D metabolomics studies may be classified as either predictive or diagnostic. In this review, we define diagnosis as the identification of a currently occurring medical condition, while prediction is defined as the identification of subjects who will develop a certain medical condition in the future. Predictive studies follow initially healthy study participants for several years in prospective study designs. During follow-up, a small number of participants will develop T2D. As a result, it is necessary to use large study cohorts with several thousands of participants. Such high demands reduce the amount of available study cohorts, and therefore most researchers have used samples from existing prospective studies including the Framingham Heart Study (FHS) [34, 35], the Cooperative Health Research in the Region of Augsburg (KORA) study [36, 37], or the European Prospective Investigation into Cancer and Nutrition (EPIC) study [36, 37] for their research. Ideally, results from one study cohort are replicated in another, independent cohort, thus ensuring the validity of the results. Diagnostic studies are usually performed using cross-sectional study designs. Such studies have the advantage of testing at a single time point with no lengthy follow-up required. Studies with as little as 73 participants have led to reproducible results [38], while other studies of this type have used sample sizes of up to 7,098 individuals [39].

An overview of the most commonly observed metabolite changes from predictive and diagnostic studies is shown in Table 1. Only metabolites found to be predictive of the onset of T2D are shown. Data on presymptopmatic individuals, those classified as having prediabetes (PD) or diagnosed as T2D, are reported. PD, which includes impaired fasting glucose, impaired glucose tolerance, and insulin resistance, may be seen as an early form of T2D; diagnosis of PD often yields similar metabolomic results as T2D (Table 1). In the following paragraphs, specific metabolites found to be predictive of T2D or PD will be discussed along with their physiological relevance.

One group of metabolites consistently linked to PD and T2D in metabolomics studies is the branched-chain amino acids (BCAA) leucine, isoleucine, and valine. Elevated levels of BCAA have been found up to 13.7 years ahead of clinical manifestation [35, 36, 40], in PD [39, 43–47] and in overt T2D [35, 37, 43, 46, 49, 50]. This association has led to the hypothesis of a causal role of these amino acids in the development of the disease [51]. However, there is growing evidence that elevated BCAA levels may reflect a state of insulin resistance that is not necessarily specific to T2D as similar signatures have been observed for cardiovascular disease, chronic kidney disease, and ischemic stroke [52]. Given this, the observed changes could also be indirect and primarily associated with insulin sensitivity rather than insulin secretion [39]. In support of this hypothesis, BCAA have a plethora of biological functions, modulating protein synthesis and turnover as part of the mammalian target of rapamycin (mTOR) pathway, facilitating glucose uptake in the liver and skeletal muscles, and also enhancing glycogen synthesis [53].

Similarly, changes in aromatic amino acids (AA) have also been linked to the development of T2D years ahead of clinical manifestation [35, 36], PD [39, 44, 46, 47], and overt T2D [35, 46, 49]. Specifically, elevated circulating levels of the AA phenylalanine and tyrosine are linked to T2D. As for BCAA, a causative effect has yet to be fully elucidated, but changes in their circulating concentrations are thought to be indirect markers of insulin sensitivity [39].

Several metabolomics studies have also found sugars, including glucose, mannose, fructose, and hexose, to be elevated 3–7 years before clinical manifestation of T2D [36, 41], in PD [37, 41, 43, 46] and overt T2D [37, 41, 43, 46, 49]. This discovery of glucose, which is one of the most standardized clinical parameters for T2D, amongst the top ranked predictive metabolites underscores its high importance for the risk assessment of T2D. Nonetheless, elevated sugar levels years ahead of overt T2D may also be caused by undiagnosed T2D amongst study participants. Additionally, metabolomics has shown an increase in *α*-hydroxybutyric acid (AHBA) up to 9.5 years ahead of T2D presentation [41, 42], in PD [41, 43, 46–48] and T2D [41, 43, 46]. It is hypothesized that increased lipid oxidation, oxidative stress [48], and enhanced glutathione synthesis [42] might explain the observed differences in AHBA.

Glycine, on the other hand, was found to be reduced 7 years before T2D [36, 37], as well as in PD [37, 38, 47] and in manifest T2D [37]. This reduction in glycine is thought to be the result of increased gluconeogenesis [36] as well as glutathione consumption driven by increased oxidative stress [54]. Also, the abundance of incompletely oxidized fuels that are excreted as urinary acylglycine conjugates could reduce glycine levels [55]. It has also been hypothesized that insulin deficiency leads to enhanced *δ*-aminolevulinate synthase 1 (ALAS-H) expression, in turn catalyzing the condensation of glycine and succinyl-CoA into 5-aminolevulinic acid, thus reducing glycine levels [37].

Several studies have also found changes in the ketones *β*-hydroxybutyrate, acetone, and acetoacetate, which originate from fatty acid oxidation. Elevated levels were found 3 years ahead of disease manifestation [41] as well as in overt T2D [41, 49]. For PD, a more complex picture is observed, where both elevated [41, 47] and reduced levels were found [39, 45]. These contradictory results are not surprising, as the role of fatty acid oxidation in the development of PD remains highly controversial. While some studies suggest that PD develops secondary to diminished fatty acid oxidation by cytosolic lipid accumulation, thereby impairing insulin signaling [56], other studies suggest PD to be characterized by excessive fatty acid oxidation, metabolic inflexibility, and coincident depletion of organic acid intermediates of the tricarboxylic acid cycle [55]. These contradictory findings from different metabolomics studies highlight the complexity of the progression to T2D and the need for further research in this area.

Other metabolites of interest include plasma glyoxylate. Glyoxylate levels were found to be increased 3 years ahead of disease onset, in PD and in overt T2D [41]. Authors of this particular study hypothesized a connection to hypertension, as glyoxylate is a substrate of alanine-glyoxylate aminotransferase-2 (AGT2), which regulates hypertension. A suppression of AGT2 activity may be associated with the development of hypertension and increased glyoxylate [41]. Likewise, 2-aminoadipic acid was reported to be elevated in individuals up to 12 years before T2D onset [34] and in PD [47]. It is hypothesized that this metabolite is part of a compensatory mechanism upregulating pancreatic insulin secretion to maintain glucose homeostasis in early insulin resistance [34]. To date, the latter two metabolites, 2-aminoadipicacid and glyoxylate, have only been identified as predictive by a single study, respectively. Further research on another T2D cohort is necessary to validate these results.

Differences and predictive profiles were also detected in lipids. For diacyl-phosphatidylcholine C32:1, elevated levels were detected years ahead of T2D [36] and also seen in PD [37] and in overt T2D [37]. Although hyperlipidemia is common in T2D [57], decreased values were detected when analyzing single lipid species. Decreased levels of sphingomyelin C16:1 and acyl-alkyl-phosphatidylcholine C34:3 were found ahead of T2D [36] and in overt T2D [37], with the latter being decreased also in PD [37].

A lipid that was found in several predictive studies is lysophosphatidylcholine (LPC) C18:2, which was reduced up to 7 years ahead of T2D [35–37], in PD [37] and in incident T2D [35, 37]. Consistently, linoleoyl-glycerophosphocholine (LGPC) was found to be reduced 9.5 years ahead of T2D [42] and in PD [47, 48]. As LGPC is one of the isomers of LPC C18:2, the association found in LPC C18:2 is likely due to the LGPC fraction contained in the total LPC C18:2 level. LGPC has been shown to increase glucose-dependent insulin secretion in a cell line derived from beta cells [42], an effect that has been linked to the orphan G-protein-coupled receptor GPR119 [58]. On the other hand, reduced LGPC levels have been linked to reduced cPLA2 transcription, which would lead to an increased glucose uptake by adipocytes via a decrease in arachidonic acid [37].

While the aforementioned studies all analyzed plasma or serum, there are a few reports employing other sample types. A study on urine found increased levels of glucose and glycine in overt T2D [23]. In saliva, 1,5-anhydroglucitol has been found to be a marker for short-term glycemic control [25], something that has already been found in blood levels of this diet-derived metabolite [49]. The value of these metabolomics based analyses in the context of T2D prediction and diagnosis has yet to be determined.

The use of prediction measures can give an insight on the usefulness of certain metabolites in disease prediction. One of the most common means to assess prediction power is receiver operating characteristic (ROC) curves, for which the area under the curve (AUC) is used as prediction measure. The AUC, also called* c-statistics*, is a value between 0 and 1, where values close to 0.5 indicate no predictive power (random classification) and values close to 1 indicate perfect prediction. The study by Floegel et al. [36] found that metabolite levels alone could predict T2D slightly better than the Diabetes Risk Score (DRS) based on risk factors such as lifestyle, diet, and anthropometry [59] with an AUC 0.849 for metabolites versus 0.847 for DRS. Adding metabolite profiles to risk prediction always enhanced the prediction, with an AUC of 0.912 for a prediction using DRS, fasting glucose, HbA_1c_, and metabolites. Similarly, Walford et al. [35] found that metabolite profiles scored slightly higher than a genetic risk score (GRS) [60], with AUCs of 0.874 versus 0.861. A combination of metabolites with GRS yielded the highest AUC of 0.880. Wang et al. [40] used a clinical model including age, BMI, and fasting glucose and found a c-statistic of 0.801, which improved to 0.805 when metabolites were included in the model. When only high-risk subjects were included in the analysis, the c-statistic increased from 0.52 to 0.66, indicating that high-risk patients benefit more from metabolic prediction than low-risk patients. Padberg and coworkers [41] found an AUC of 0.82 when using only metabolites compared to an AUC of 0.79 when using only fasting glucose levels to predict T2D. In a second cohort, the value improved from 0.83 to 0.86, respectively, for the prediction of PD. Wang et al. [34] used a prediction based on age, sex, fasting glucose, and BMI, resulting in a c-statistic of 0.91, which improved to 0.92 when including 2-aminoadipic acid. The net reclassification improvement (NRI), an alternative measure for prediction power [61] for these models, increased from 0.22 to 0.49. In the study by Wang-Sattler and coworkers [37], the AUC of a model containing age, sex, BMI, physical activity, alcohol intake, smoking, systolic BP, and HDL was 0.742 and increased to 0.754 when including metabolites. When including HbA_1c_, fasting glucose, and fasting insulin into the model, the values were 0.818 and 0.828, respectively. Ferrannini et al. [42] found an AUC of 0.762 when including familial diabetes, sex, age, and BMI. This value increased to 0.790 when including metabolites. In a second study, the values were 0.766 and 0.783, respectively. The objective interpretation of these numbers is hampered by the use of different combinations of clinical parameters to estimate T2D risk. While some studies use only fasting glucose, some use complex combinations of classical risk factors. This makes it impossible in some cases to determine whether metabolite signatures really improve T2D prediction when compared to the best available classical risk factors. Still, some studies use an extensive number of classical risk factors and obtain improved results when adding metabolite profiles to their prediction.

This selective overview shows some of the drawbacks of metabolomics studies: the results often differ between studies and at times can even contradict each other. One reason for this may be that different analytical platforms measure different subsets of the metabolome, often with little overlap between different platforms. Other reasons include different characteristics of the observed populations (age and genetic background) as well as the use of different data analysis techniques. Granting all this, T2D risk prediction years ahead of overt disease has been shown to be feasible using metabolomics. As mentioned, a set of 12 metabolites or metabolite classes has been identified as predictive for T2D (Table 1), a substantial number that could possibly be integrated into standard, bench top laboratory tests for diagnostic purposes.

## 3. Prognosis: T2D Complications

Apart from diagnosing or predicting the onset of PD or T2D, another important field for metabolomics research is prognosis, eluding disease course and outcome after its onset. The course and outcome of PD and T2D are to a large proportion driven by common complications including cardiomyopathy, nephropathy, peripheral neuropathy, and retinopathy. Unfortunately, in this area, little metabolomics research has been performed on human subjects, even though improved prognosis may enable early interventions, alleviate disease burden, and facilitate cost-effective treatments.

It is estimated that 60–70 percent of individuals with T2D have some form of cardiac dysfunction or cardiovascular disease. As a result, individuals with T2D are twice as likely as healthy controls to have heart disease or stroke, making cardiovascular disease (CVD) the number one complication of diabetes [62]. Still, CVD risk is often poorly controlled in T2D patients [63].

Wu et al. [64] used metabolomics to investigate specific cardiovascular risk factors including high blood pressure, nonalcoholic fatty liver disease, and coronary heart disease in a group of T2D patients. Their goal was to find biomarkers indicating one or more of these T2D comorbidities. Unfortunately, this study only analyzed metabolic differences between these three risk factors and combinations thereof and did not include a control group without CVD risk factors. Although the study could show notable metabolic differences between these diseases and their combinations, the authors state that the value of the results is limited as they were not able to recruit a matching control group without T2D complications. Likewise, another study failed to predict coronary artery disease in 190 T2D patients within 4 years of follow-up [65].

For diabetic nephropathy, slightly more research has been performed. Sharma et al. [66] compared urine metabolites of diabetes patients with and without chronic kidney disease. Although both type 1 and type 2 diabetes patients were enrolled in the study, only T2D cases were used in the screening cohort. It was found that 13 urinary metabolites were significantly reduced in chronic kidney disease patients. Interestingly, 12 out of these 13 metabolites were found to be connected to mitochondria, and as such the authors suggested impaired mitochondrial metabolism in diabetic kidney disease. However, reduced urinary levels may also stem from reduced kidney function and thus not impaired mitochondrial function per se [66].

In a rare prospective study, Niewczas and colleagues [67] analyzed the plasma of 40 T2D patients who developed end-stage renal disease within 8–12 years of follow-up and compared it to a matched control group. Uremic solutes and acylcarnitines were significantly increased prior to end-stage renal disease, hinting at early reductions in renal function that did not show up in standard renal function tests based on glomerular filtration rate estimated from serum creatinine levels. A set of AA and their derivatives showed decreased levels; notably, BCAA and aromatic AA as well as AHBA were significantly decreased, suggesting enhanced mitochondrial AA *β*-oxidation [67]. Of interest, these metabolites were decreased rather than increased as usually seen for both the prediction and diagnosis of T2D (Table 1). The results of this study have not yet been replicated in an independent cohort, but another study comparing T2D with and without diabetic nephropathy also found reduced levels of BCAA and aromatic AA [68].

Finally, a study has been conducted on diabetic retinopathy, which is the leading cause of blindness and visual impairment among working-age persons in developed countries [69], finding reduced levels of galactitol and ascorbic acid in vitreous humor [70]. However, vitreous humor is a hard-to-collect sample type, which limits the application of this kind of analysis. Additionally, the control group in this study was not made up of healthy individuals but of patients with a macular hole. This might further limit the general applicability of the results.

All in all, the study of T2D-associated complications is still underrepresented in field metabolomics research, especially regarding prospective studies on human samples. Further investigations similar to that of Niewczas et al. [67], who prospectively analyzed metabolomic changes ahead of end-stage renal disease in T2D patients, have the potential to precipitate early interventions based on individual risk assessments.

## 4. Future Directions: Translational Efforts

Translation into clinical practice is defined as enabling the use of results from basic metabolomics research to enhance diagnosis, prediction, prognosis, and therapy. In the context of metabolomics, this is achieved by defining a predictive or diagnostic profile for a specific population (age and sex) under a defined set of conditions (fed and fasted). Basic research metabolomics studies typically measure hundreds of metabolites, an approach that is not feasible or cost-effective for large-scale application. Additionally, laboratory equipment used for metabolomics studies is expensive and not readily available at the point of care. For these reasons, only a small subset of relevant metabolites that can be assessed using standard equipment or assays could be incorporated into a clinical routine. These profiles would be assessed in a clinical biochemistry setting. Although much more work needs to be done, such measures have the potential to be quick, cost-effective, and relatively easy to interpret. Identification of a high probability of T2D risk in a presymptomatic patient could inform physicians of the need for early testing, intensive intervention, and continued monitoring. This approach has the potential to be far more effective compared to treating full-blown T2D, where irreversible damage may have already occurred. Thus, the translation of metabolomics research into clinical application may lead to informed decision-making in the realm of personalized medicine.

To date, work translating metabolomics findings into clinical application has been limited, and most studies have focused on T2D prediction rather than T2D comorbidities. There may also be value in combining metabolomics data with other molecular and clinical datasets. A good example of this integration can be found in Walford et al. [35], who analyzed a combination of 62 genetic and 9 metabolic biomarkers for the purpose of predicting T2D risk. Results showed that biomarkers such as the BCAA isoleucine, the aromatic AA phenylalanine and tyrosine, triacylglycerides, phosphatidylcholines, and lysophosphatidylcholines were found to be predictive up to 13.4 years before the onset of disease. When metabolomic, genotypic, and clinical data were analyzed together there was a significant rise in predictive performance, suggesting that these datasets can be used to compliment or even validate each other. Of note, the authors of this study state that they may not have used the most ideal combination of biomarkers.

Investigators Varvel and colleagues [71] have also made significant advancements in translating metabolomics research into clinical application. Specifically, they assessed a panel of 19 blood-based biomarkers, including metabolites such as glucose and AHBA, proteins, and antibodies. Based on these values, they analyzed T2D risk in 1,687 patients presenting for risk assessment. Patients belonged to an obese high-risk cohort, with 48 percent of patients meeting the criteria for metabolic syndrome and one-third having been previously diagnosed with T2D and/or hypertension. Screening by classical clinical parameters classified 929 patients (55.1%) as normoglycemic. When classifying using biomarker analysis, 766 out of these normoglycemic patients (82.5%) were classified as “at-risk.” This indicates that a large amount of high-risk patients is missed when relying solely on classical clinical parameters. The classification results were presented to the patient and their physician and included treatment considerations, with the final treatment decision made exclusively by the treating physician. During care, guided by biomarker testing, a significant amount of patients initially classified as “high normal” or prediabetic had improved their values. Although these results are promising and represent an example of how informed decision-making can be based on metabolomics data, a randomized study design will have to be employed in order to authenticate these results.

It is clear that further research on translating metabolomics results into clinical applications is necessary. In order for this to be successful, a robust set of relevant biomarkers must be defined, considering also that the metabolites have to be measured cost-effectively in a standard clinical environment. In any case, it may be feasible for metabolomic biomarkers to be used in addition to established clinical tests for T2D and its comorbidities. Day-to-day variations in the metabolomic profile, which occur due to differences in diet, physical activity, and so on, may be minimized when analyzing serial time points of the same individual to create an average metabolic profile [72]. Additionally, insights from metabolomics studies may be the basis for genome-wide association studies (GWAS) [73–75], possibly identifying genetic backgrounds of the observed effects and further strengthening the personalized aspects of diagnosis and treatment.

## 5. Conclusions

The field of metabolomics is still relatively young and exists at a basic research level, where the issues of measurements and data analysis often require novel solutions. Moreover, metabolomics data often lacks standardization in its reporting, thus making it difficult to compare results from independent studies. In addition, differences in protocols and instrumentation can yield varied sets of detectable metabolites, often with little overlap to other designs. Even when similar methods are used, different statistical approaches might lead to contradictory results.

Despite these potential limitations, metabolomic studies have the potential to determine a unique set of metabolites that are predictive of both PD and T2D, often years or decades ahead of disease onset. This powerful information may shed light on disease development and may improve patients' health, as shown in the pilot study by Varvel et al. [71], who used a set of metabolites in a clinical setting to inform physicians on T2D risk factors. Although there is much work left to do, the evidence of metabolomics benefitting T2D care makes its clinical application inevitable.

## Figures and Tables

**Table 1:** Metabolites found to predict type 2 diabetes in advance of onset as well as their response to prediabetes and overt type 2 diabetes.

Metabolite class	3–14 years ahead							Prediabetes										Type 2 diabetes						
BCAA^a^		↑	↑		↑					↑	↑	↑	↑	↑	↑			↑	↑	↑	↑	↑	↑	
Aromatic amino acids^b^		↑	↑		↑					↑		↑		↑	↑			↑			↑	↑		
Sugars^c^			↑			↑		↑			↑			↑		↑			↑	↑	↑	↑		↑
*α*-Hydroxybutyrate						↑	↑				↑			↑	↑	↑	↑			↑	↑			↑
Glycine			↓	↓				↓	↓						↓				↓					
Ketone bodies^d^						↑				↓			↓		↑	↑						↑		↑
Glyoxylate						↑										↑								↑
2-Aminoadipic acid	↑														↑									

Diacyl-PtC C32:1			↑					↑											↑					
SM C16:1			↓																↓					
Acyl-alkyl-PtC C34:3			↓					↓											↓					
Lyso-PtC C18:2		↓	↓	↓				↓										↓	↓					
Linoleoyl-GPC							↓								↓		↓							

Reference	[34]	[35]	[36]	[37]	[40]	[41]	[42]	[37]	[38]	[39]	[43]	[44]	[45]	[46]	[47]	[41]	[48]	[35]	[37]	[43]	[46]	[49]	[50]	[41]
Sample type	#P	##	#S	FS	FP	NP	FP	FS	FS	FS	FP	FS	FP	FS	FP	NP	FP	##	FS	FP	FS	FP + FS	FP	FP
Instrumentation	LC-MS	LC-MS	LC-MS	LC-MS	LC-MS	GC, LC, GC-MS	LC-MS	LC-MS	GC-MS, LC-MS	NMR	LC-MS	GC-MS, LC-MS	MS	GC-MS, LC-MS	LC-MS	GC, LC, GC-MS	GC-MS, LC-MS	LC-MS	LC-MS	LC-MS	GC-MS, LC-MS	GC-MS, LC-MS, NMR	LC-MS	GC, LC, GC-MS
*N* (cases)	376 (188)	1622 (206)	3082 (800)	876 (91)	378 (189)	366 (179)	2580 (151)	1104 (238)	73 (28)	7098 (781)	2204 (192)	140	82	111 (24)	1623 (191)	366 (179)	399 (140)	1622 (206)	957 (91)	2204 (115)	111 (27)	100 (40)	182 (105)	366 (179)
Age [y]	56 ± 9	56.67 ± 8.94	54.7 ± 7.3	65.4 ± 10.3	57 ± 8	55.23	52 ± 12	65.4 ± 10.3	77.7 ± 3.9	31 ± 3	60.01 ± 12.40	38–60	13.76 ± 0.26	42.9 ± 10.6	68 ± 7	55.23	45 [11]^*^	56.67 ± 8.94	65.4 ± 10.3	63.00 ± 9.61	42.9 ± 10.6	67.7 ± 7.2	53.6 ± 8.99	55.23
Sex	f/m	f/m	f/m	f/m	f/m	f/m	f/m	f/m	f/m	f/m	f	f/m	f/m	f/m	f/m	f/m	f/m	f/m	f/m	f	f/m	m	f/m	f/m
Other	12 years ahead	13.5 years ahead	7 years ahead	7 years ahead	12 years ahead	6 years ahead	9.5 years ahead		Elderly functionally limited		Twin study		Obese adolescents							Twin study				

Each arrow represents a single, independent study showing the respective metabolite to be significantly changed. Upward arrow: upregulated versus control; downward arrow: downregulated. ^a^Branched-chain amino acids including valine, leucine, and isoleucine. ^b^Aromatic amino acids include phenylalanine, tryptophan, and tyrosine. ^c^Sugars include glucose, hexose, mannose, and fructose. ^d^Ketone bodies including acetoacetic acid, acetone, and *β*-hydroxybutyric acid. ^*^Median [interquartile range]. BCAA: branched-chain amino acids, FP: fasting plasma, FS: fasting serum, GPC: glycerophosphocholine, NP: nonfasted plasma, NS: nonfasted serum, PtC: phosphatidylcholine, SM: sphingomyelin, #P: plasma with unknown fasting status, #S: serum with unknown fasting status, and ##: unknown sample type with unknown fasting status.
